# No secret hiding place? Absence of SARS-CoV-2 on the ocular surface of 1145 hospitalized patients in a pandemic area

**DOI:** 10.1007/s00417-021-05086-3

**Published:** 2021-01-29

**Authors:** Alexander C. Rokohl, Rafael S. Grajewski, Philomena A. Wawer Matos, Hannah-Leah Koch, Felix Dewald, Florian Klein, Gerd Fätkenheuer, Clara Lehmann, Claus Cursiefen, Ludwig M. Heindl

**Affiliations:** 1 Department of Ophthalmology, Faculty of Medicine and University Hospital of Cologne, University of Cologne, Kerpener Strasse 62, 50937, Cologne, Germany; 2 Laboratory of Experimental Immunology, Institute of Virology, Faculty of Medicine and University Hospital Cologne, University of Cologne, Cologne, Germany; 3 Department I of Internal Medicine, Division of Infectious Diseases, University of Cologne, Cologne, Germany; 4 German Center for Infection Research (DZIF), Partner Site Bonn-Cologne, Cologne, Germany; 5 Center for Molecular Medicine Cologne (CMMC), University of Cologne, Cologne, Germany

**Keywords:** COVID-19, Conjunctival swaps, Coronavirus, SARS-CoV-2, Nasopharyngeal swaps, Ocular surface

## Abstract

**Objectives:**

The aims of this study were to evaluate the isolated prevalence of real-time reverse transcriptase-polymerase chain reaction (RT-PCR)–confirmed SARS-CoV-2 on the ocular surface without systemic infection in hospitalized asymptomatic patients and to determine the risk for ophthalmologists and medical staff to be infected by prescreened asymptomatic patients in a tertiary eye care center.

**Methods:**

In this prospective, observational study, bilateral swaps of the conjunctiva in the lower fornices as well as nasopharyngeal swaps were collected in 1145 hospitalized asymptomatic patients of a tertiary eye care center. Real-time reverse transcriptase-polymerase chain reaction (RT-PCR) analysis was performed for each swap to evaluate the prevalence of SARS-CoV-2. Demographic data and potential risk factors for an isolated infection of the ocular surface were noted.

**Results:**

Two thousand two hundred eighty-eight (99.9%) of all 2290 tested eyes had negative results in the RT-PCR analysis of the conjunctival swabs. One patient had bilateral false-positive results in the conjunctival swabs. None of the 1145 patients had any positive RT-PCR-confirmed result in the nasopharyngeal swabs.

**Conclusions:**

The risk for an isolated conjunctival viral activity in patients with a negative nasopharyngeal swab-based RT-PCR seems to be absent or extremely low, suggesting no need to perform additional conjunctival swabs in patients with negative nasopharyngeal swabs. Furthermore, the risk of a work-related SARS-CoV-2 infection due to direct contact with preselected asymptomatic patients in an eye care center is very low, especially when additional hygiene standards and safe distances are respected carefully. This might reassure medical staff and reduce the fear of SARS-CoV-2 infection.

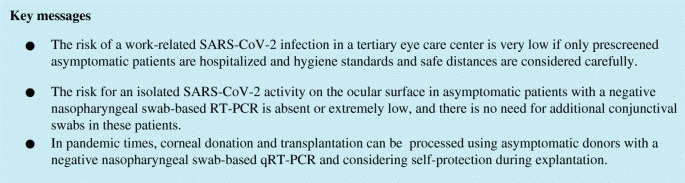



## Introduction

The coronavirus disease 19 (COVID-19) pandemic, caused by the *severe acute respiratory syndrome-*related coronavirus (SARS-CoV-2), is a public health emergency of tremendous international relevance [1–6]. SARS-CoV-2 spreads via human-to-human and seems to be transmitted mainly through respiratory droplets and contact with infected persons, less commonly via smear infection [1, 2, 4, 5]. Furthermore, there seems to be a significant risk for ophthalmologists and medical staff to be infected with SARS-CoV-2 during the ophthalmological examination, treatment, or care due to the close proximity to infected (but often still asymptomatic) patients or colleagues [1, 2, 4, 7–9]. In addition, this potentially high risk seems to affect the mental health of ophthalmologists and the medical staff resulting in anxiety and depression symptoms [10].

Previous studies reported the presence of the entrance receptor of SARS-CoV-2, the angiotensin-converting enzyme 2 (ACE 2), on the ocular surface and that a significant proportion of SARS-COV-2-positive patients demonstrates unspecific conjunctivitis [1, 6, 11–13]. Furthermore, SARS-CoV-2 was detected in ocular tissues, in tears, and in conjunctival secretions of COVID-19 patients [3, 4, 6, 11, 14–16]. Therefore, a potential transmission and infection with SARS-CoV-2 by ocular secretions seem to be possible.

However, until now, it is not investigated, whether SARS-CoV-2 might exist isolated on the ocular surface without systemic infection. Furthermore, the risk of an infection with SARS-CoV-2 for ophthalmologists and the medical staff under pandemic conditions is also not determined, yet. Therefore, the purpose of this prospective, observational study was to evaluate the isolated prevalence of real-time reverse transcriptase-polymerase chain reaction (RT-PCR) confirmed SARS-CoV-2 on the ocular surface and in nasopharynx in hospitalized asymptomatic patients and to determine the risk and risk factors for ophthalmologists to be infected by asymptomatic prescreened patients during work in a tertiary eye care center in Germany.

## Patients and methods

The study was conducted at the Department of Ophthalmology, University of Cologne, Germany, in adherence to the tenets of the Declaration of Helsinki and its later amendments. Institutional Review Board approval was obtained (20-1161). Inclusion criteria were hospitalizing for any reason at the Department of Ophthalmology of the University Hospital Cologne, Germany. All patients who were hospitalized in the University Eye Hospital Cologne between the 22nd of April and 11th of July were asked to participate. After explanation of the nature of the study, informed consent was obtained from all participants. None of the patients did report symptoms indicating COVID-19 including fever, cough, common cold, loss of smell or taste, headache, body aches, shortness of breath, sore throat, or fatigue. Demographic data of all participants including age, gender, and ethnicity were recorded. Furthermore, the reason for hospitalization, application frequency of topical eye medication per day, the use of antiviral or immunosuppressive topical medication, and contact lens use were queried as potential risk factors for SARS-CoV-2 infection of the ocular surface. In addition, the use of antiviral or immunosuppressive systemic medication, the use of systemic ACE inhibitors, and if the participants already had RT-PCR-confirmed COVID-19 were evaluated as potential risk factors for systemic or topical infection.

Afterward, body temperature was measured using a daily calibrated infrared ear thermometer (Genius 2™, Cardinal Health Germany 507 GmbH, Norderstedt, Germany). Body temperature was graded as normal (≤ 37.4 °C) or elevated (≥ 37.5 °C). Afterward, nasopharyngeal swaps and bilateral swaps of the conjunctiva in the lower fornices were collected. Nasopharyngeal and conjunctival swabs were placed each in a universal transport medium (UTM) (Copan Diagnostics, Murrieta, USA (USA)) and nucleic acids were extracted from UTM using a MagNA Pure 96 (Roche, Basel, Switzerland). SARS-CoV-2-RNA was detected by real-time RT-PCR confirming the presence of the viral E and RdRP genes (TIB molbiol, Berlin, Germany) or E and S genes (Altona Diagnostics GmbH, Hamburg, Germany), respectively, using a LightCycler 480 or LightCycler 480 II (Roche, Basel, Switzerland) [17]. SPSS version 26.0 for Mac (SPSS, Inc., Chicago, IL) was used for all statistical analyses.

## Results

Out of the 1145 hospitalized participants, 544 (47.5%) were males and 601 (52.5%) females with a mean age of 59 ± 22 years (range, 0–95 years). One thousand one hundred fourteen (97.3%) were European, 5 (0.4%) African, 3 (0.3%) Asian, and 23 (2.0%) were from the Middle East. 11.4% (*n* = 131) of the patients used ACE 2 inhibitors, 40 (3.5%) systemic immunosuppression medication, and 14 (1.2%) systemic antiviral medication. None of the 1145 patients reported RT-PCR-confirmed COVID-19 before hospitalization.

Main reasons for hospitalization included corneal surgery (24.4%; *n* = 279 patients); conduct of 24-h eye pressure measurements without surgery (23.2%; *n* = 266); cataract surgery (16.3%; *n* = 187); retinal or vitreous surgery (10.7%; *n* = 122); strabismus surgery (8.3%; *n* = 95); glaucoma surgery (6.9%; *n* = 79); and eyelid (3.2%; *n* = 37), tumor (3.0%; *n* = 34), and lacrimal surgery (1.7%; *n* = 20).

Contact lenses were used in 54 of 2290 (2.4%) eyes. Topical medications were used in 1330 (58.1%) eyes with a mean frequency of 3.7 per day. Topical immunosuppressive medication was used in 405 (17.7%) eyes and antiherpetic eye drops in 18 eyes (0.8%). During the evaluation time, the use of face masks was mandatory for all patients and hospital staff at all times.

None of the 1145 patients (100%) showed an elevated body temperature > 37.4 °C or any symptoms of COVID-19. In addition, none of the 1145 patients (100%) had any positive RT-PCR-confirmed result in the nasopharyngeal swabs. Although most of the patients had a very high risk for self-inoculation of SARS-CoV-2 in the eyes by using several times a day topical eye medication, 2288 (99.9%) of all 2290 tested eyes had negative results in the RT-PCR analysis of the conjunctival swabs. One hospitalized patient had bilateral positive RT-PCR results in the conjunctival swabs, but this result was putatively wrongly positive because it could not be confirmed, neither in the second analysis of these positive swaps nor in further conjunctival swabs. Furthermore, this patient did not develop any symptoms of COVID-19 or conjunctivitis at a later point.

## Discussion

First of all, this study investigated the potential isolated prevalence of SARS-CoV-2 on the ocular surface and adds therefore significant new knowledge which is very important and should be considered by ophthalmologists in these pandemic times. In addition, the present study determines the risk for a work-related SARS-CoV-2 infection of ophthalmologists and medical staff by asymptomatic prescreened patients for the first time and has therefore several very important implications under the pandemic situation in Germany at the time analyzed:

(i) The risk of a work-related infection due to direct contact with ocular fluids in an eye care setting with prescreened asymptomatic patients is very low. This might reassure ophthalmologists and medical staff and reduce the fear of a work-related SARS-CoV-2 infection.
(ii) The risk for an isolated SARS-CoV-2 activity on the ocular surface in asymptomatic patients with a negative nasopharyngeal swab-based RT-PCR seems to be absent or extremely low. Therefore, there seems to be no need to perform additional conjunctival swabs in prescreened asymptomatic patients with negative nasopharyngeal swabs.
(iii) Reasons for the very low prevalence of positive results both in the nasopharyngeal and conjunctival swaps might be the sensitivity of these swabs or the relatively low prevalence rate of active cases in Cologne during the study period. On the other hand, only asymptomatic patients without any symptoms and without elevated body temperature were screened with nasopharyngeal and conjunctival swabs at the Department of Ophthalmology of the University Hospital Cologne. This preselection might reduce the likelihood of a positive result significantly. However, the results of this study should be confirmed in similar trials with higher prevalence rates of active cases during the study period.
(iv) The results of our study imply also a virtually absent risk for contamination of ocular surface fluids and human corneal donor tissue if the donor was asymptomatic (and a conjunctival and/or nasopharyngeal swab-based qRT-PCR analysis was negative). These new findings are of very high relevance since there is considerable uncertainty under which conditions cornea donation should be conducted, on how to preselect corneal donors, and on how corneal tissue should be processed and tested in pandemic times.

In summary, based on the very low prevalence of SARS-CoV-2 infection in preselected asymptomatic hospitalized patients, the risk a work-related infection with SARS-CoV-2 in an tertiary eye care center seems to be low, especially when additional hygiene standards and safe distances are respected carefully [18, 19]. In pandemic times, corneal donation and transplantation can be processed using asymptomatic donors with a negative nasopharyngeal swab-based qRT-PCR and considering self-protection with protective equipment during corneal explantation.

## Data Availability

The data that support the findings of this study are available on request from the corresponding author. The data are not publicly available due to privacy or ethical restrictions.
